# Kardiovaskuläre Risikoevaluation in der Behandlung des Hodgkin-Lymphoms – auf dem Weg zur individualisierten Planung?

**DOI:** 10.1007/s00066-023-02051-3

**Published:** 2023-02-07

**Authors:** Michael Oertel, Hans Theodor Eich

**Affiliations:** Klinik für Strahlentherapie – Radioonkologie, Universitätsklinikum Münster, Albert-Schweitzer-Campus 1, Gebäude A1, 48149 Münster, Deutschland

## Hintergrund

Das Hodgkin-Lymphom (HL) weist in frühen Stadien durch die Kombination von Systemtherapie und konsolidierender Radiotherapie (RT) ein langfristiges krankheitsfreies Überleben von > 90 % auf [1–4]. Aufgrund der exzellenten Langzeitprognose versuchen moderne Studien eine isoeffektive Therapiedeeskalation, die insbesondere den Einsatz der RT kritisch evaluiert [1–4]. Insofern ist eine dezidierte Risikoevaluation der Therapie notwendig. Die britischen Kollegen liefern mit einer Analyse zur langfristigen kardiovaskulären Morbidität und Mortalität im Rahmen des RAPID Trial wichtige Daten für eine differenzierte Diskussion [3, 5].

## Methode/Patientengut

Der RAPID Trial schloss Patienten im Ann-Arbor-Stadium I/IIA ohne Bulkbefall ein und sah zunächst eine Chemotherapie mit drei Zyklen Doxorubicin, Bleomycin, Vinblastin und Dacarbazin (ABVD) mit nachfolgender Positronen-Emissions-Tomographie(PET)-Kontrolle vor [3]. PET-negative Patienten (Deauville-Score 1–2) wurden randomisiert zwischen einer Involved-field-Radiotherapie (IFRT) mit 30 Gray (Gy) und keiner weiteren Therapie, während PET-positive Patienten obligat einen weiteren Zyklus ABVD und eine IFRT erhielten.

Das Risiko wurde anhand der RT-Dosis am gesamten Herzen, den Herzklappen, dem linken Ventrikel, den Aa. carotis communes sowie der gegebenen Anthrazyklindosis über kohortenbasierte Risikomodelle ermittelt. Eine alters- und geschlechtsadjustierte Gruppe der gesunden Bevölkerung aus dem UK diente als Referenz. In die Analyse wurden 183 PET-negative Patienten und 129 PET-positive Patienten eingeschlossen, für die in 78,7 % bzw. 79,8 % dosimetrische Daten vorlagen.

## Ergebnisse

Die mittlere Herzdosis (MHD) betrug 4,0 Gy (0,3 Gy ohne bzw. 7,8 Gy mit mediastinalem Befall) bzw. 4,5 Gy (0,4 Gy ohne bzw. 10,4 Gy mit mediastinalem Befall) für die PET-negativen bzw. PET-positiven Patienten. Für die Karotiden wurden mittlere Dosen zwischen 20 und 21,8 Gy angegeben (28,2 –29,6 Gy bei mediastinalem Befall). Konsekutive Risikoangaben wurden nur für das PET-negative Kollektiv berichtet. Für die kardiovaskuläre 30-Jahres-Mortalität bestand ein Basisrisiko der Vergleichskohorte von 3,52 % mit zusätzlichen 0,94 % durch die anthrazyklinhaltige Chemotherapie und 0,56 % durch die RT (0,01–6,79 %; Median: 0,26 %). Für 67 % der Patienten betrug das Mortalitätsrisiko < 0,5 %. Nach RT waren v. a. ischämische Herzerkrankung (0,36 %) und Schlaganfälle (0,14 %) maßgeblich für die Mortalität. Insgesamt differierte das individuelle Mortalitätsrisiko nach 30 Jahren zwischen 0,002 und 6,55 % im Hinblick auf kardiale Erkrankungen (mittlerer Wert: 0,42 %; 0,79 % mit bzw. 0,05 % ohne Mediastinalbeteiligung) und zwischen 0,008 und 1,12 % für Schlaganfälle (mittlerer Wert: 0,14 %). Analog zur Betrachtung der Mortalität wurden 30-Jahres-Morbiditätsdaten errechnet mit einem mittleren Risiko von 35,8 % (7,7–86,8 %). Das Basisrisiko der Normalbevölkerung belief sich auf 22,9 %, während 6,7 % bzw. 6,2 % auf ABVD-Chemotherapie bzw. IFRT entfielen. Das mediane individuelle Risiko nach RT betrug 3,61 % (0,31–31,09 %) und wurde erneut vor allem durch ischämische Herzerkrankung und Schlaganfälle getragen.

## Schlussfolgerung der Autoren

Für die meisten Patienten ist eine RT risikoarm und vorteilhaft. Bei hoher Dosisexposition bieten sich „fortgeschrittene Techniken“ oder der Verzicht auf eine RT an. Insgesamt plädieren die Autoren für eine individualisierte risikoadaptierte Planung.

## Kommentar

Aus strahlentherapeutischer Sicht leiten sich mehrere Erkenntnisse ab:

- Das mit einer RT verbundene kardiovaskuläre Morbiditäts- und Mortalitätsrisiko ist niedrig und liegt (meist) sogar unter dem der ABVD-Chemotherapie.
- Die gezeigten Schwankungen der Werte (Faktor 100+) sind immens und hängen wesentlich von der Zielvolumenlage ab (Mediastinalbefall? Lage untere Feldgrenze?).
- Zu Recht wird von den Autoren auf das Armamentarium der modernen Radioonkologie verwiesen (Review [6]): Bestrahlung in tiefer Inspiration, intensitätsmodulierte Techniken oder Protonen-RT. Technikbedingt kann die RT-Belastung mediastinaler Organe beim HL differieren und eine Schonung ermöglichen [7].
- Durch die detaillierte Konturierung kardialer Substrukturen (Kammern, Klappen, Erregungsleitsystem) und Berücksichtigung bei der Risikoermittlung ergibt sich eine differenzierte Weiterentwicklung der reinen MHD-Betrachtung.

Zu berücksichtigen sind folgende Einschränkungen:

- Das Risikomodell adjustierte nicht für relevante Faktoren wie Rauchen und Diabetes.
- Nur für 66 % der Patienten lagen individuelle CT-Aufnahmen und DICOM-Dateien vor, sodass eine stringente Dosisanalyse erfolgen konnte. Bei 28 % mussten die RT-Felder anhand von digital rekonstruierten Radiographien oder Simulationsfilmen erst nachgebildet und dann auf eine Referenz-CT übertragen werden. Auch war bei 30 % der Patienten das Herz nicht bzw. inkomplett auf der Planungs-CT abgebildet.
- Das Felddesign der IFRT wurde mittlerweile weiterentwickelt. Die dosimetrische Analyse der HD17-Studie der Deutschen Hodgkin-Studiengruppe (GHSG) zeigte eine mittlere Herzdosis von 13,1 Gy (Median der verfügbaren DVH) mit ebenfalls hoher Spanne (0,5–30,4 Gy; [8, 9]). Im Vergleich zwischen IFRT und der kleineren Involved-node-RT ergaben sich keine signifikanten Unterschiede (MHD: 14,4 Gy vs. 12,4 Gy; *p* = 0,691).
- Die RAPID-Studie inkludierte Patienten, die nach Definition der GHSG den Risikogruppen „early-favorable“ oder „early‑unfavorable“ angehören, was die Bewertung erschwert. Zuletzt konnte für HD16 („early-favorable“) ein prognostischer Vorteil durch die zusätzliche RT dargelegt werden, für HD17 („early-unfavorable“) jedoch nicht [1, 4].

### Schlussfolgerungen

Die vorgelegte Analyse unterstreicht die Bedeutung einer individualisierten risikobasierten Planung in der strahlentherapeutischen Behandlung des HL. Sie zeigt, dass für den überwiegenden Teil der Patienten die RT eine sichere und effektive Behandlung ist, sodass ein generalisierter Verzicht auf diese wichtige Modalität nicht angezeigt ist.

*Michael Oertel, Hans Theodor Eich, Münster*
